# Supplementary material for: Regulatory T cells trigger effector T cell DNA damage and senescence caused by metabolic competition
Source: Nat Commun. 2018 Jan 16;9:249. doi: 10.1038/s41467-017-02689-5 (PMC5770447; doi:10.1038/s41467-017-02689-5)
Supplement: Supplementary file 1 — Supplementary Information [file 41467_2017_2689_MOESM1_ESM.pdf]

Supplementary Figure 1

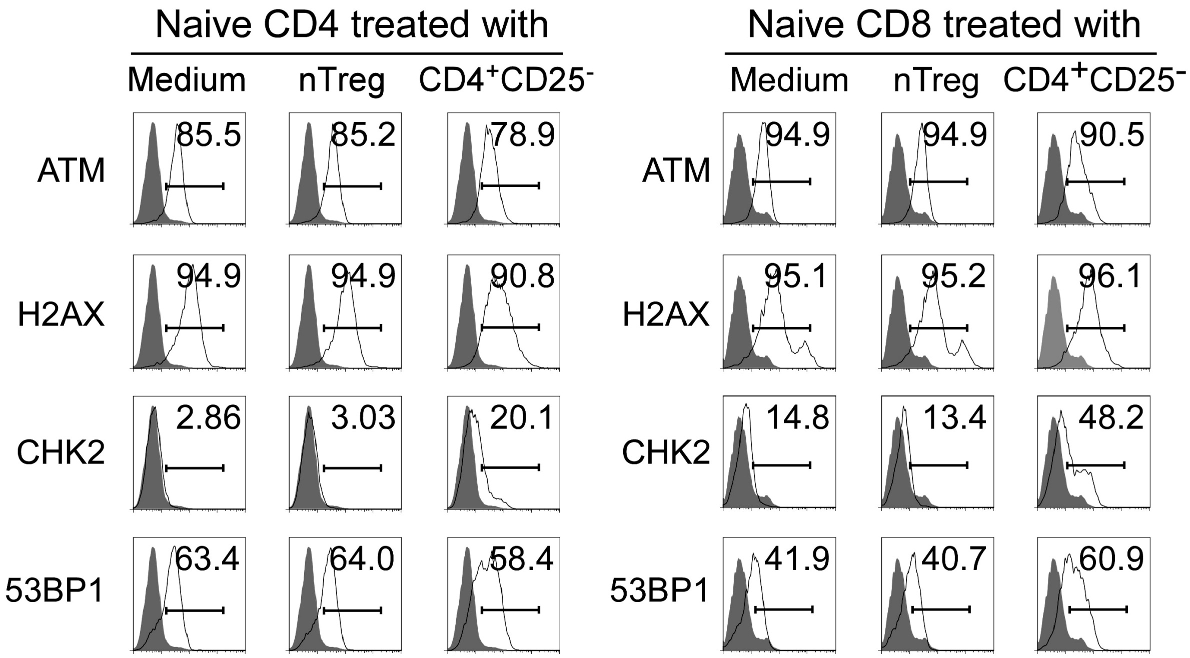

Supplementary Figure 1. Treatment with nTreg cells did not alter the expression levels of total ATM and other associated molecules H2AX, 53BP1 and CHK2 in naïve CD4$^+$ and CD8$^+$ T cells. Anti-CD3 activated naïve CD4$^+$ and CD8$^+$ T cells were treated with nTreg or CD4$^+$CD25$^-$ effector T cells (control). The ATM, H2AX, 53BP1 and CHK2 protein expression levels in treated naïve CD4$^+$ and CD8$^+$ T cells were analyzed by the flow cytometry.

Supplementary Figure 2

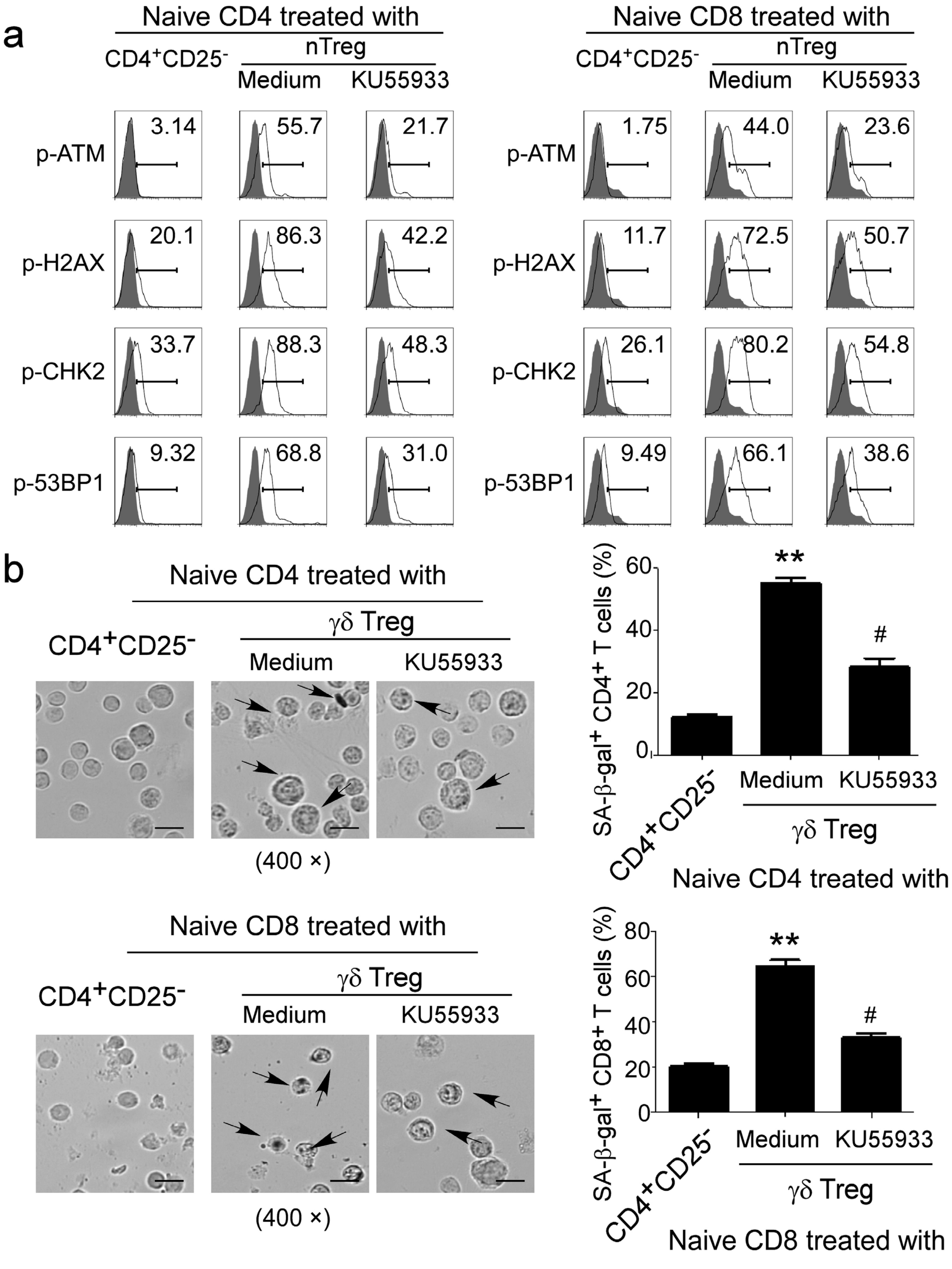

Supplementary Figure 2. Human Treg-induced T-cell senescence involves the DNA damage response in responder T cells.

(a) Pretreatment of responder T cells with the ATM inhibitor KU55933 significantly prevented up-regulation of the phosphorylated ATM and other associated molecules H2AX, 53BP1 and CHK2 in pre-activated naïve $CD4^+$ and $CD8^+$ T cells treated with nTreg cells. Anti-CD3-activated $CD4^+$ and $CD8^+$ T cells were pretreated with or without KU55933 (10 μM) for 1 day, and then co-cultured with nTreg cells for 3 days. The p-ATM, p-H2AX, p-53BP1, and p-CHK2 expression in treated naïve $CD4^+$ and $CD8^+$ T cells were analyzed by the flow cytometry. **(b)** Pretreatment of naïve $CD4^+$ and $CD8^+$ T cells with ATM inhibitor KU55933 significantly prevented T cell senescence induced by γδ Treg cells. Anti-CD3 activated T cells were pretreated with or without KU55933 (10 μM) for 1 day, and then co-cultured with γδ Treg cells for 3 days. SA-β-Gal expression in naïve $CD4^+$ and $CD8^+$ T cells was determined with SA-β-Gal staining. The SA-β-Gal positive T cells were identified with dark blue granules as indicated by the arrows. Scale bar: 20 μm. Data shown in the right panels are mean ± SD from three independent experiments. **$p<0.01$, compared with the medium only and KU55933 treatment groups by paired t-test.

Supplementary Figure 3

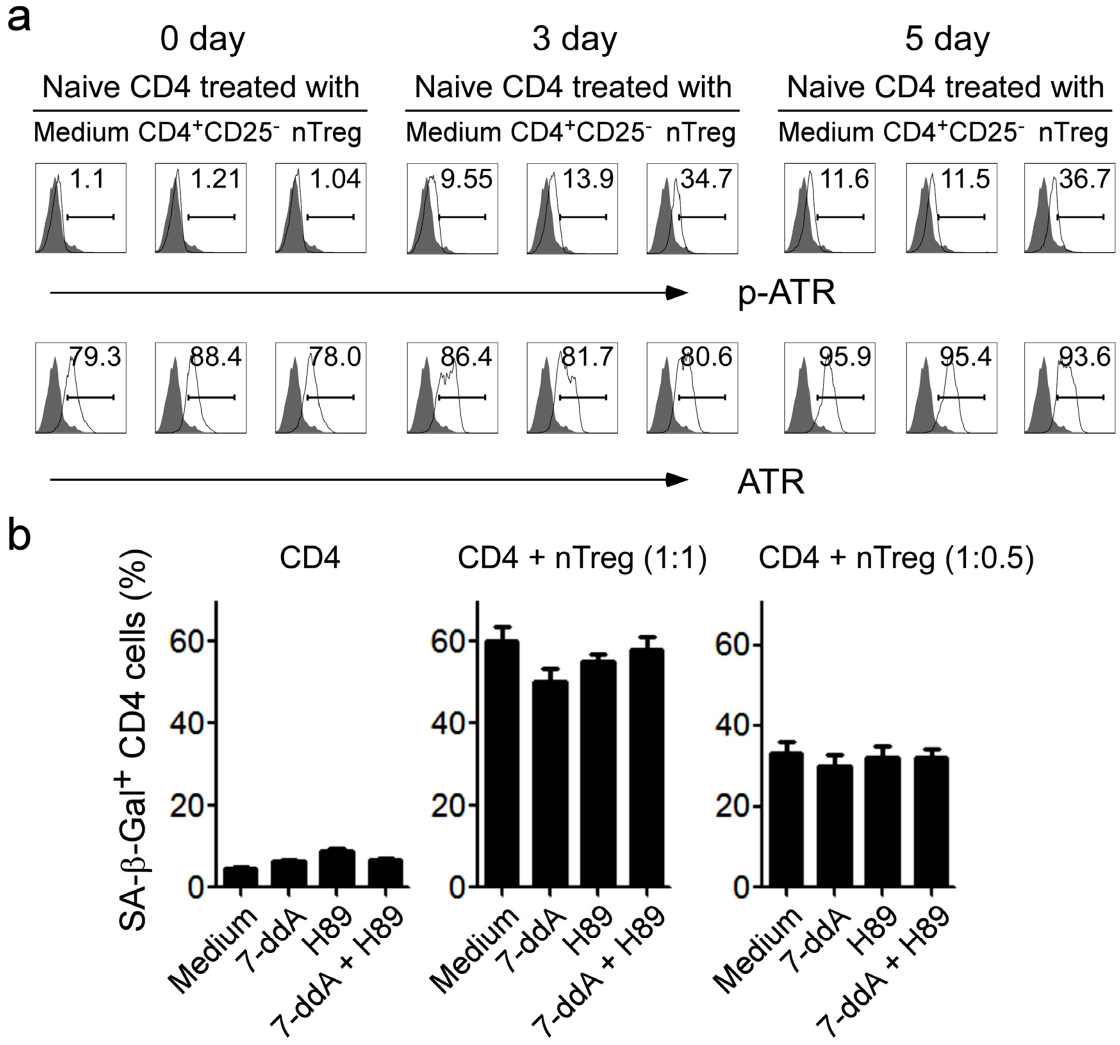

Supplementary Figure 3. Human Treg-induced T-cell senescence involves the ATR activation in responder T cells.

(a) Phosphorylated activation of ATR in naïve CD4$^+$ T cells treated with human nTreg cells. Anti-CD3 activated naïve CD4$^+$ T cells were treated with nTreg or CD4$^+$CD25$^-$ effector T cells (control) for 3 days. The p-ATR and total ATR expression in treated naïve CD4$^+$ T cells were analyzed by the flow cytometry. **(b)** cAMP was not involved in the induction of T cell senescence mediated by human nTreg cells. Treatment of Treg cells with 2', 5'-dideoxyadenosine (7ddA, an inhibitor for adenylate cyclase) and H89 (an inhibitor for PKA) cannot prevent induction of senescence in responder T cells mediated by nTreg cells. CFSE labeled CD4$^+$ T cells were co-cultured with or without nTreg cells for 3 days at ratio of 1:1 and 1:0.5 in the presence or absence of inhibitors 7ddA (40 μM), H89 (10 μM), or combination, and then purified by FACS sorting. The separated CD4$^+$ T cells were determined with SA–β-Gal expression.

Supplementary Figure 4

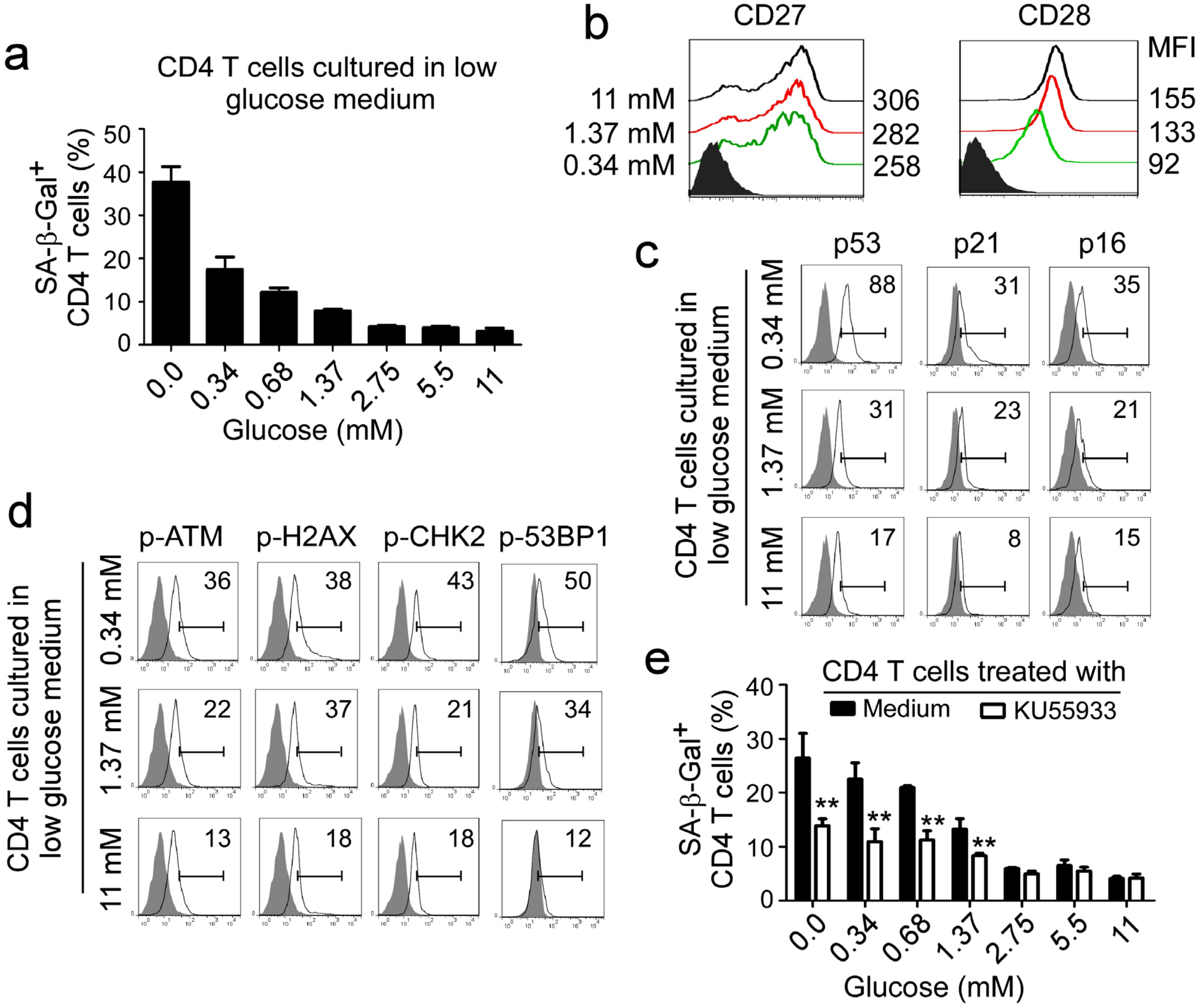

Supplementary Figure 4. Low concentrations of glucose cause T cell DNA damage and senescence.

(a) Significantly increased SA-β-Gal^{+} T cell populations were induced in naïve CD4^{+} T cells cultured in the medium with low concentrations of glucose. Anti-CD3-activated naïve CD4^{+} T cells were cultured with different concentrations of low glucose for 5 days. Data shown are mean ± SD from CD4^{+} T cells from three individual healthy donors. Normal medium with 11 mM glucose served as a control. SA-β-Gal expression in CD4^{+} T cells was determined. **(b)** Down-regulation of CD28 and CD27 in naïve CD4^{+} T cells induced by the culture medium with low concentrations of glucose. Cell treatment and procedure were the same as in (a). CD27 and CD28 expression in treated naïve CD4^{+} T cells were analyzed by the flow cytometry. Mean Fluorescence Intensity (MFI) was calculated as shown in the right. **(c)** Increased expression of cell cycle regulatory molecules p53, p21 and p16 in naïve CD4^{+} T cells cultured with low concentrations of glucose medium. Cell treatment and procedure were the same as in (a). The p53, p21 and p16 expression in treated CD4^{+} T cells were analyzed by the flow cytometry. **(d)** Phosphorylated activation of ATM and other associated molecules H2AX, 53BP1 and CHK2 in naïve CD4^{+} T cells cultured with low concentrations of glucose medium. Cell treatment and procedure were the same as in (a). The p-ATM, p-H2AX, p-53BP1, and p-CHK2 expression in cultured naïve CD4^{+} T cells were analyzed by the flow cytometry. **(e)** ATM specific inhibitor KU55933 significantly prevented CD4^{+} T-cell senescence induced by the low glucose condition. Anti-CD3 pre-activated naïve CD4^{+} T cells were cultured with different concentrations of glucose medium in the presence or absence of the ATM specific inhibitor KU55933 (10 μM) for 5 days. SA-β-Gal expression in CD4^{+} T cells was determined. Data shown are mean ± SD from three independent experiments. $^{**}p<0.01$, compared with the medium only group using paired t-test.

Supplementary Figure 5

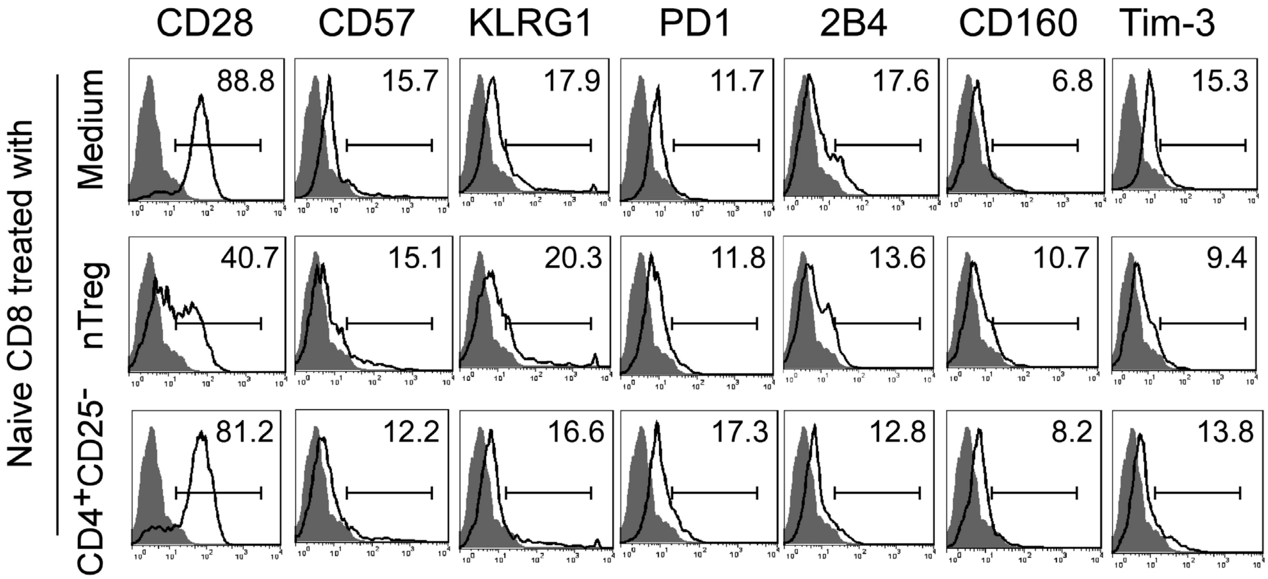

Supplementary Figure 5. Human Treg cells induce senescence but not exhaustion in responder naive CD8$^+$ T cells.

Anti-CD3-preactivated naïve CD8$^+$ T cells were co-cultured with nTreg or CD4$^+$CD25$^-$ effector T cells (control) for 5 days, and then expression markers on treated T cells were determined by the flow cytometry analyses.

Supplementary Figure 6

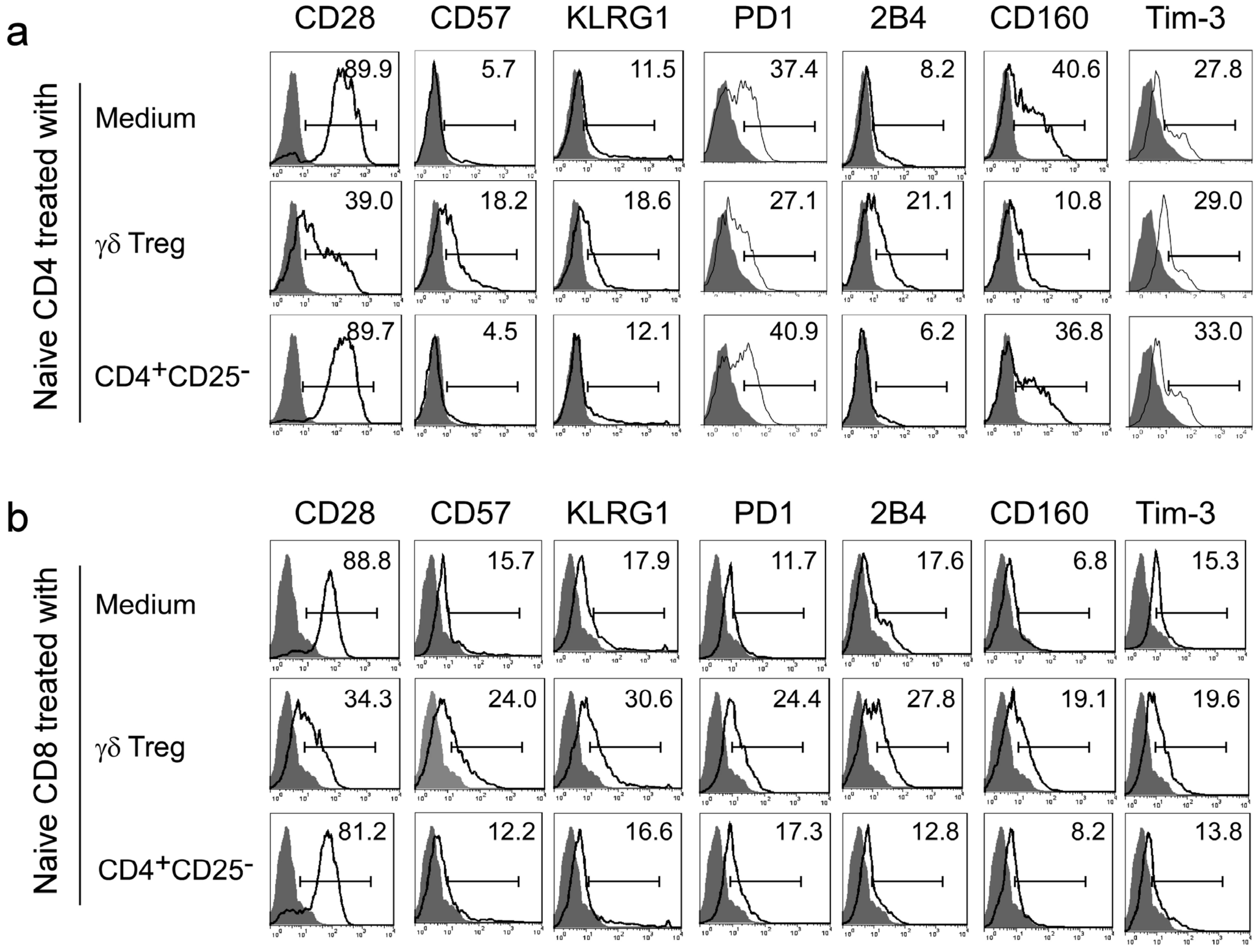

Supplementary Figure 6. Senescent T cells induced by human γδ Treg cells are not exhausted T cells.

(a) and (**b)** Human γδ Treg cell treatment did not promote the expression of exhaustion markers in senescent $CD4^+$ (in a) and $CD8^+$ (in b) T cells during the senescence development. Anti-CD3-preactivated naïve $CD4^+$ and $CD8^+$ T cells were co-cultured with γδ Treg or $CD4^+CD25^-$ effector T cells (control) for 5 days, and then expression markers on treated T cells were determined by the flow cytometry analyses.

Supplementary Figure 7

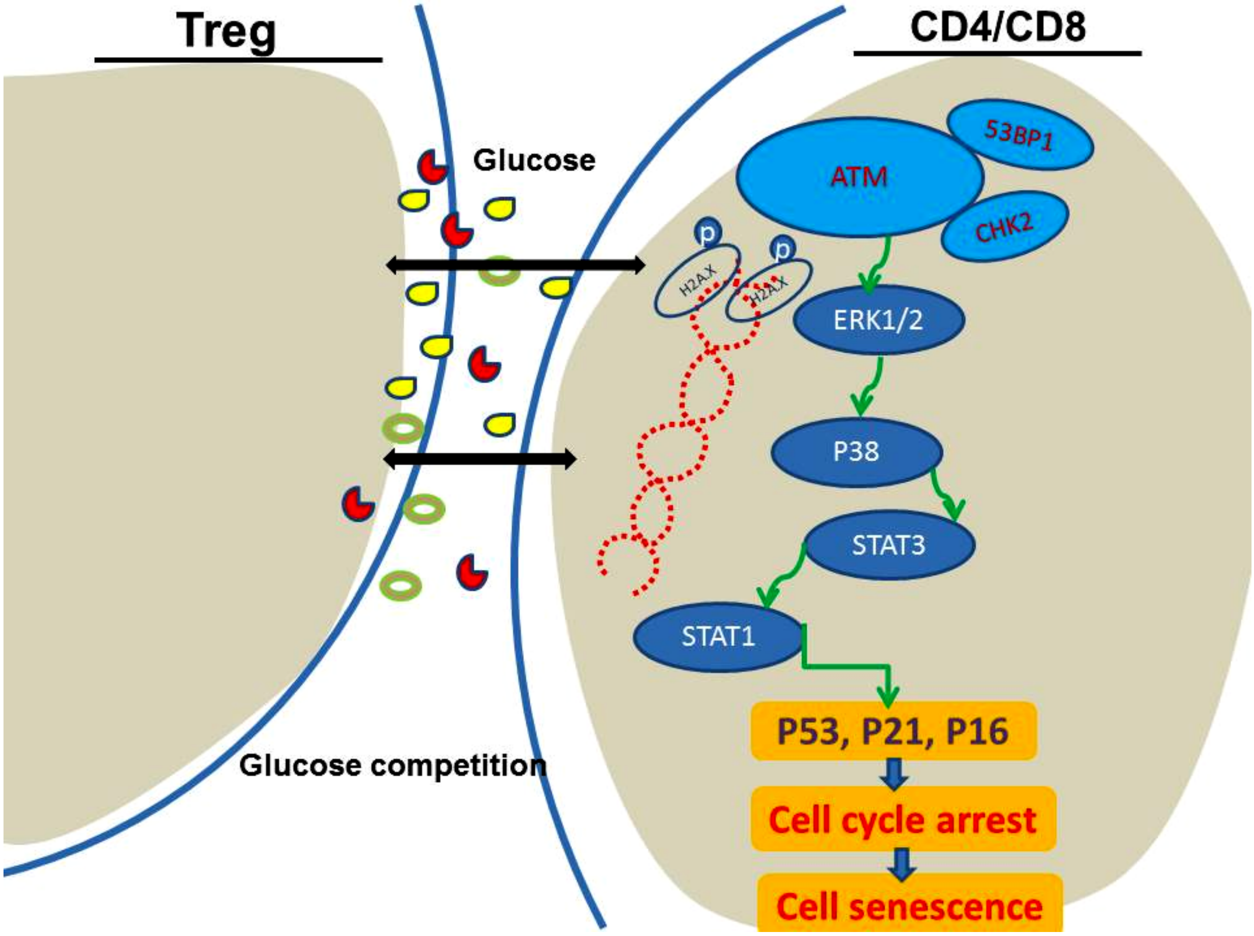

Supplementary Figure 7. Diagram of the senescence induction in responder $CD4^+$ and $CD8^+$ T cells mediated by human Treg cells.

Human Treg cells directly suppress responder T cell proliferation and initiate the ATM-associated DNA damage response through glucose competition, resulting in the cell cycle arrest and senescence in responder T cells. The ATM-associated DNA damage and cell senescence processes are molecularly controlled by MAPK ERK1/2 and p38 signaling functionally cooperating with transcription factors STAT1 and STAT3.

Supplementary Figure 8

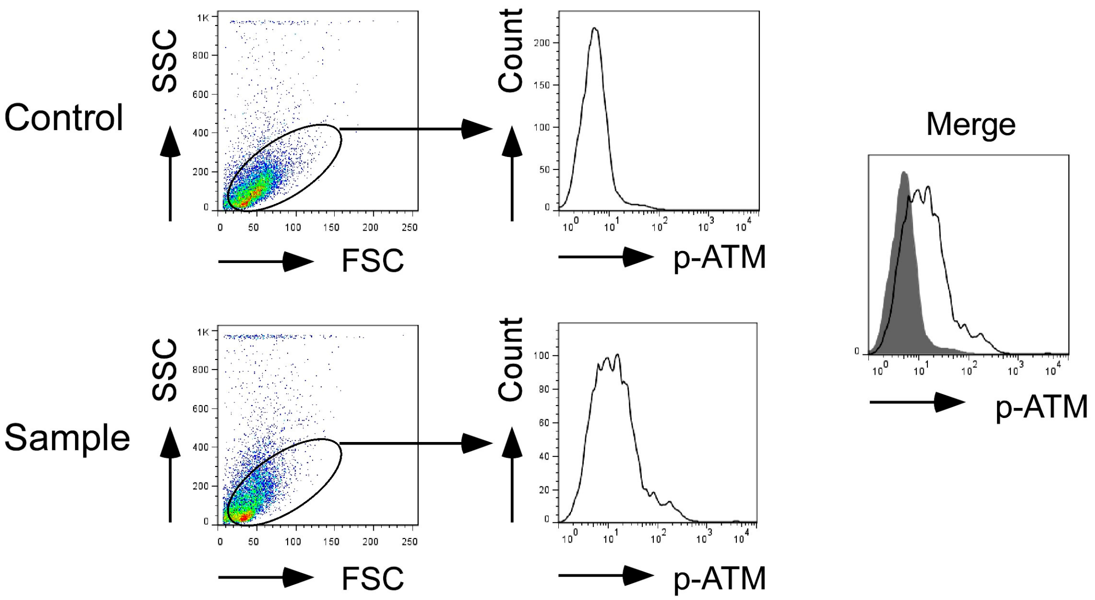

Supplementary Figure 8. Diagram of the gating strategy utilized for the flow cytometry analyses of expression markers on T cells.

This gating strategy analyzing p-ATM expression on T cells as a representative was utilized for all T cell markers determined by the flow cytometry after surface staining or intracellular staining with anti-human specific antibodies conjugated with PE or Alexa Flour488. Those analysis results are shown in Figures. 1, 2, 3, 6, 8 and Supplementary Figures 1-6.

Supplementary Table 1. Primers used for real-time quantitative RT-PCR

Genes	Primers
IL-1β Forward	ACAGATGAAGTGCTCCTTCCA
IL-1β Reverse	GTCGGAGATTCGTAGCTGGAT
IL-2 Forward	GTCACAAACAGTGCACCTAC
IL-2 Reverse	CCCTGGGTCTTAAGTGAAAG
IL-6 Forward	GTAGCCGCCCCACACAGA
IL-6 Reverse	CATGTCTCCTTTCTCAGGGCT
CAPASE3 Forward	CGGATGGGTGCTATTGTGAGGCG
CAPASE3 Reverse	GGCCACGGATACACAGCCACAG
CTLA4 Forward	GTAATTGATCCAGAACCGTGCCCAG
CTLA4 Reverse	TTCTGGCTCTGTTGGGGGCAT
DGKA Forward	GGCTCGATGCCTAAGATGGGGA
DGKA Reverse	AGAGGCATCCACGCCAATAGAGA
EGR2 Forward	CGGAGTGGCCGGAGATGGCA
EGR2 Reverse	ACGGGAGCAAAGCTGCTGGG
FYN Forward	GCGGGGAGAGGACCATGTGAGT
FYN Reverse	AGAGGCAGGACTGGTCTTTTTCCA
ITCH Forward	ATCCCCGCACAGGAAAATCTGCC
ITCH Reverse	GTGGCATGGCCAGTTGCTGAC
LCK Forward	GGAGCTGGGACCCCCTATTTTAGCT
LCK Reverse	TGCCCTTGCCATCCAGTGGGA
PGAM1 Forward	AATCCCAGTCGGTGCCGCAT
PGAM1 Reverse	CATAGCCAGCATCTCGTAGCGCC
PDCD1 Forward	GTGGGGCTGCTCCAGGCATG
PDCD1 Reverse	GGTGGCGTTGTCCCCTTCGG
PIM1 Forward	GCGGCTTCGGCTCGGTCTAC
PIM1 Reverse	GTGCCATTAGGCAGCTCTCCCC
RAB10 Forward	AAGGAGAACAGATTGCAAGGGAGCA
RAB10 Reverse	GCTCTTCCAGCCTGTCACGCC
SOCS2 Forward	CCACCTGTCTTTGCCGCGGT
SOCS2 Reverse	CCCAGTACCATCCTGTCTGACCGA
TLE4 Forward	TCCTCTTCGGGGTCATTAAAGCCAA
TLE4 Reverse	CTGATGCGGTGCCGGGTGTC
TOB1 Forward	TGCTGCCACCAAGTTCGGCTC
TOB1 Reverse	AGCCAAGCCCATACAGAGAGTGCA
TRAF5 Forward	ACTAACGGTTCTGAAGCGGAATGG
TRAF5 Reverse	TGGGGGTTGTGAAGCACCGAG
Glut1 Forward	AAT GGC TCC GGT ATC GTC AAC
Glut1 Reverse	GCT CAG ATA GGA CAT CCA GGG TA
Glut3 Forward	GCTCTCTGGGATCAATGCTGTGT
Glut3 Reverse	CTTCCTGCCCTTTCCACCAGA

Supplementary Table 2. Genes used for the comparison in senescent T cells and anergic T cells

Gene	Description
APOE	Antagonizes posttranslational events in mitogen-activated T cells
ARF6	ADP-ribosylation factor 6
BAX	BCL2-associated X protein
CASP3	caspase 3, apoptosis related cysteine protease
CD7	CD7 molecule, cell differentiation antigen 7
CDKN1B	cyclin-dependent kinase inhibitor 1B (p27, Kip1)
CTLA4	cytotoxic T-lymphocyte-associated protein 4
DGKA	diacylglycerol kinase, alpha 80kDa
EGR2	early growth response 2
FYN	FYN oncogene related to SRC, FGR, YES
GBP3	guanylate binding protein 3
ITCH	itchy E3 ubiquitin protein ligase homolog
ITGB2	integrin, beta 2 (complement component 3 receptor 3 and 4 subunit)
LCK	lymphocyte-specific protein tyrosine kinase
PDCD1	programmed cell death 1
PGAM1	phosphoglycerate mutase 1
PIM1	pim -1 oncogene (proviral integration site 1)
RAB10	RAB10, member RAS oncogene family ,G-protein signaling
RNF128	ring finger protein 128
SLC3A2	solute carrier family 3 (activators of dibasic and neutral amino acid transport), member 2
SOCS2	suppressor of cytokine signaling 2
TLE4	transducin-like enhancer of split 4 (E(sp1) homolog, Drosophila)
TNFRSF1B	tumor necrosis factor receptor superfamily, member 1B
TNFSF9	tumor necrosis factor (ligand) superfamily, member 9
TOB1	transducer of ERBB2, 1
TRAF5	TNF receptor-associated factor 5
